# Nationwide Etoricoxib Injection Clinical Experience (NICE) 2.0: Assessing Real-World Effectiveness and Tolerability of Etoricoxib Injection in Acute Pain Management in Both Inpatient and Outpatient Settings

**DOI:** 10.7759/cureus.90116

**Published:** 2025-08-14

**Authors:** SB Uday Kumar, Shubh Mehrotra, Amulya Singh, Tanay Prabhoo, Sameer Muchhala, Vidhya Natarajan

**Affiliations:** 1 Orthopedics, Parmanand Deepchand (PD) Hinduja Sindhi Hospital, Bangalore, IND; 2 Orthopedics, Lovee Shubh Hospital, Lucknow, IND; 3 Orthopedics, Akshat Seva Sadan, Patna, IND; 4 Spine Surgery, Lilavati Hospital and Research Centre, Mumbai, IND; 5 Medical Affairs, Zydus Healthcare Limited, Mumbai, IND

**Keywords:** acute pain management, etoricoxib, inpatient settings, intramuscular (im) formulation, outpatient settings, visual analog scale

## Abstract

Background: Effective acute pain management remains a challenge. Etoricoxib, a selective COX-2 inhibitor, is known for its favorable safety profile compared to traditional NSAIDs. While oral etoricoxib is well-established for pain management, the intramuscular (IM) formulation remains underrepresented in the published literature and warrants further clinical investigation.

Objectives: This study aims to assess the effectiveness of IM etoricoxib (90 mg/mL) in reducing acute pain severity in inpatient and outpatient settings and to evaluate its tolerability.

Materials and methods: In this retrospective, multicenter, observational cohort study, medical records of 1,018 patients treated at the discretion of doctors across 296 centers in India were reviewed. Pain was assessed on the 10-point Visual Analog Scale (VAS). In outpatient settings, VAS score recordings were taken at baseline and at 30- and 60-minute post-injection intervals. In inpatient settings, recordings were taken at baseline, 30 minutes, 60 minutes, 6 hours, 12 hours, and 24 hours. Adverse events were documented.

Results: The study included 1,018 patients, 229 (22.5%) inpatients and 789 (77.5%) outpatients. The primary indication for IM etoricoxib injection was acute back pain (272, 26.7%). Pain reduction, measured via VAS, showed significant results. In outpatients, VAS scores reduced by 61.6% at 60 minutes post-injection (p < 0.001). In inpatients, pain reduction was sustained over 24 hours, with a cumulative reduction of 69.45% at 24 hours (p < 0.001). The need for rescue analgesics was minimal (2.9%). Safety analysis revealed no adverse events in 992 (97.4%) of patients, with mild pain at the injection site in 338 (33.2%).

Conclusion: IM etoricoxib demonstrated rapid and sustained pain relief with excellent tolerability across diverse patient populations.

## Introduction

Acute pain is a major global health concern, affecting approximately one in five adults worldwide [1]. In hospital settings, it remains highly prevalent, with 37-53% of patients reporting pain during hospitalization and surgical patients having more than twice the odds of experiencing it [2]. Such pain has a significant impact on daily functioning, with nearly 43% of hospitalized patients reporting moderate to severe interference with general activity [2]. Despite advances in pain management, the problem persists; for example, surveys in China reported pain prevalence rates of 31.6% in 2011 and 24.3% in 2021 [3]. Beyond the immediate discomfort, inadequately managed acute pain can transition into chronic pain, leading to long-term consequences such as sleep disturbances, impaired daily activities, and diminished quality of life [1,4,5]. These observations underscore the ongoing need for effective, rapid-onset, and well-tolerated analgesic options across clinical settings.

Nonsteroidal anti-inflammatory drugs (NSAIDs) are widely used for managing acute pain due to their analgesic and anti-inflammatory properties. NSAIDs work by inhibiting cyclooxygenase (COX) enzymes, which are crucial for prostaglandin production [6,7]. There are two types: non-selective NSAIDs, which inhibit COX-1 and COX-2, and selective COX-2 inhibitors [8].

Etoricoxib is an NSAID used primarily for pain management. Unlike non-selective NSAIDs, etoricoxib selectively targets COX-2, responsible for pain and inflammation, while sparing COX-1, which protects the gastric lining and regulates platelet function [9-11]. This results in effective analgesia with better gastrointestinal safety [8].

Etoricoxib's oral formulation is well-established for acute and chronic pain conditions, such as osteoarthritis, rheumatoid arthritis, and postoperative pain. Its efficacy in reducing pain and inflammation, combined with a lower risk of gastrointestinal issues than non-selective NSAIDs, makes it a safer option for long-term use [8].

When rapid pain relief is desired and oral administration is not feasible, intramuscular (IM) etoricoxib offers a practical alternative. Clinical observations with IM etoricoxib suggest a rapid onset of action, with patient-reported pain relief evident within 60 minutes. The known pharmacokinetic properties of etoricoxib, including a large volume of distribution (~120 L) and a terminal half-life of approximately 24 hours, contribute to sustained peripheral analgesic effects following administration. Additionally, the IM route bypasses the gastrointestinal tract and first-pass metabolism, which can be advantageous in patients unable to tolerate oral intake [12].

This IM formulation, developed using advanced solubilization (Ad-Sol) technology, provides a stable, water-soluble etoricoxib for injection. Ad-Sol technology ensures stability and minimizes injection site irritation [12]. IM etoricoxib fills a critical gap in parenteral NSAID options, enhancing pain management strategies.

To our knowledge, Shetty et al.'s Nationwide Etoricoxib Injection Clinical Experience (NICE) was the first real-world study on IM etoricoxib, establishing its effectiveness and tolerability in outpatient settings [12]. With NICE 2.0, the authors aimed to expand the evidence base to inpatients, offering a broader assessment of IM etoricoxib’s role in pain management.

## Materials and methods

This study employed a retrospective, observational cohort design to assess the effectiveness and tolerability of IM etoricoxib (90 mg/mL) in real-world clinical settings. The injections were administered at the treating physicians' discretion, per etoricoxib's prescribing information. After obtaining ethics committee approval, we evaluated medical records from both outpatient and inpatient settings over six months, from November 2022 to April 2023. The evaluation was conducted at 296 study sites across India, and 1,018 patients met the eligibility criteria for inclusion in the study. Patients were eligible for inclusion if they (1) were treated with IM etoricoxib (90 mg/mL) for acute pain during the study period and (2) had complete and retrievable medical records, including baseline and follow-up pain scores. There were no restrictions based on age, gender, or comorbidities. Patients with incomplete documentation of pain assessment or treatment response were excluded from the analysis. Relevant data were extracted and entered into standardized case record forms for analysis.

Data collected included patients' demographic details, specifically age and gender, and the clinical indications for which etoricoxib injection was prescribed. Pain was assessed using a 10-point Visual Analog Scale (VAS), where 0 represented no pain, scores of 1-3 indicated mild pain, 4-6 denoted moderate pain, and 7-10 signified severe pain. In outpatient settings, VAS scores were recorded at baseline and 30 and 60 minutes after administering the IM etoricoxib injection. In inpatient settings, VAS scores were recorded at baseline, 30 minutes, 60 minutes, 6 hours, 12 hours, and 24 hours post-injection. Comorbidities and adverse events were obtained from physician notes and patient records. Physician-reported outcomes on pain relief were assessed using a categorical scale documented in the case record forms, with physicians rating the patient’s response as “better,” “equal,” or “poor” compared to other NSAID injections. No inter-group variability in measurement methods existed, as all assessments followed the same standard protocols.

The primary outcome measure was the effectiveness of IM etoricoxib, assessed by the reduction in pain at various time points following its administration. Secondary outcomes included the evaluation of tolerability, determined by any adverse events reported post-injection, including pain at the injection site.

Statistical analysis

Following the predefined statistical analysis plan, data were analyzed using descriptive statistics to summarize patient demographics, baseline characteristics, and treatment outcomes. Continuous variables like age and VAS scores were reported as means ± standard deviations. In contrast, categorical variables, such as comorbidities and adverse effects, were expressed as frequencies and percentages. One-sample t-tests were used to assess the significance of changes in VAS scores from baseline at each time point post-injection. A p-value of <0.05 was considered statistically significant. Missing data were minimal and were reported where relevant throughout the results.

Ethical considerations

The study protocol was reviewed and approved by the Royal Pune Independent Ethics Committee (approval number: ECR/45/Indt/MH/2013/RR-19) at its meeting held on 1st June 2024. As this was a retrospective analysis of anonymized patient data, the requirement for informed consent was waived. Patient confidentiality was maintained throughout the study, following the protection regulations. All data were anonymized prior to analysis, and no identifiable patient information was collected or reported. The Declaration of Helsinki and Good Clinical Practice guidelines were used to conduct the study. Given the real-world, multicenter observational design, no additional institutional ethics approvals from participating hospitals were required beyond the central ethics approval, as all data were derived from routine clinical care without any interventions outside standard practice. Patients and the public were not involved in the design, conduct, reporting, or dissemination plans of this retrospective study, as it was based on anonymized data from existing medical records.

## Results

Of the 1,018 patients included in the analysis, 22.5% (n=229) were classified as inpatients, while 77.5% (n=789) were outpatients. This included 637 males (62.6%) and 381 females (37.4%). The average age of the study population was 47.42 ± 12.17 years (range: 13-90 years).

Among the chief complaints reported by patients, back pain emerged as one of the most common issues, particularly acute back pain and low back pain. Data on chief complaints were unavailable for 157 patients (15.4%). Among the 861 patients with available data, low back pain was reported by 152 patients (22 inpatients, 9.6%, and 130 outpatients, 15.1%), accounting for 17.6% of the cases with data. Acute back pain was reported by 87 patients (5 in inpatients, 2.2%, and 82 in outpatients, 9.5%).

Inpatients frequently presented with low back pain (22, 9.6%) and fractures (8, 3.5%), followed by postoperative pain (5, 2.2%) and injuries (6, 2.6%). Outpatients exhibited higher frequencies of low back pain (130, 15.1%) and acute back pain (82, 9.5%), along with knee pain (61, 7.1%), osteoarthritis (20, 2.3%), knee arthritis (16, 1.9%), muscle sprain (13, 1.5%), severe low back pain (13, 1.5%), ankle pain (11, 1.3%), and shoulder pain (11, 1.3%).

This analysis underscores the prevalence of back pain and musculoskeletal complaints across inpatients and outpatients. Low back pain is the most frequently reported condition among patients with available data.

The primary indications for etoricoxib injection, as reported by the treating doctors, are summarized in Table 1. Acute back pain was the most frequently reported indication, accounting for 272 cases (26.7%). This was followed by knee arthritis (234, 23%), postoperative cases (189, 18.6%), and fractures (206, 20.2%). Other indications included muscle strain or sprain (149, 14.6%), post-traumatic pain (136, 13.3%), back injuries (123, 12.1%), and torn ligaments (64, 6.3%).

**Table 1:** Primary indications for etoricoxib injection as reported by the treating doctors Data is expressed as the number of patients (percentage). n: number of patients

Primary indications	n (%)
Acute back pain	272 (26.7)
Knee arthritis	234 (23)
Fracture	206 (20.2)
Postoperative cases	189 (18.6)
Muscle strain/sprain	149 (14.6)
Post-traumatic pain	136 (13.3)
Back injury	123 (12.1)
Torn ligament	64 (6.3)

This distribution reflects the broad application of etoricoxib injections in managing pain related to musculoskeletal conditions, particularly those involving acute back pain, joint-related conditions, and trauma.

Comorbidity analysis

Among the 601 patients with available data for diabetes, 290 (48.3%) had diabetes, while 311 (51.7%) did not. Inpatients accounted for 40 cases (6.7%) of diabetes and 86 cases (14.3%) without diabetes, whereas outpatients had 250 cases (41.6%) of diabetes and 225 cases (37.4%) without diabetes.

Data were available for 613 patients with hypertension. Hypertension was reported in 296 cases (48.3%), and 317 cases (51.7%) did not. Inpatients represented 53 cases (8.6%) with hypertension and 79 cases (12.9%) without hypertension, while outpatients accounted for 243 cases (39.7%) with hypertension and 238 cases (38.8%) without hypertension.

For CVD, data were available for 427 patients. CVD was present in 15 cases (3.5%), and 412 cases (96.5%) did not. Among inpatients, 3 cases (0.7%) had CVD, while 106 cases (24.8%) did not. Among outpatients, 12 (2.8%) had CVD, and 306 (71.7%) did not.

This analysis demonstrates that diabetes and hypertension were prevalent among the study population, with nearly half of the patients with available data affected. CVD, however, was reported in a much smaller proportion of patients.

Pain reduction in outpatients

**Figure 1:**
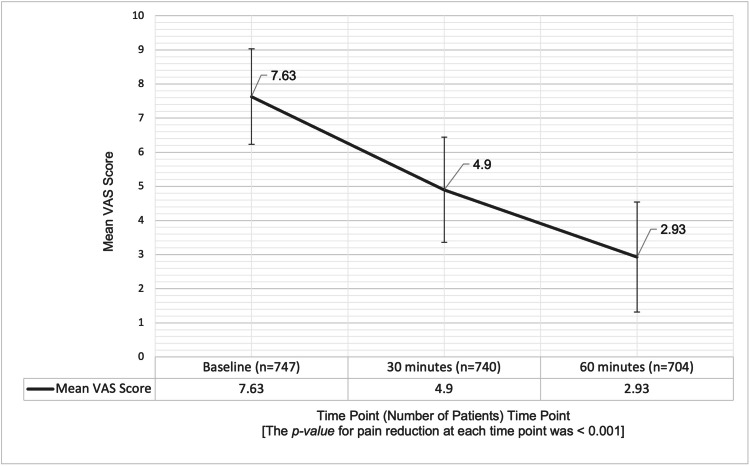
Mean VAS scores over time in outpatients following IM etoricoxib administration Error bars represent SD. The p-value for pain reduction at each time point versus baseline was <0.001. IM: intramuscular, SD: standard deviation, VAS: Visual Analog Scale

VAS measured pain reduction in outpatients at three time points: baseline (0 minutes), 30 minutes, and 60 minutes after treatment. A total of 747 patients had available data at baseline, with a mean VAS score of 7.63 ± 1.40, indicating severe pain at baseline (Figure 1).

At 30 minutes, the mean VAS score decreased significantly to 4.90 ± 1.54 (p < 0.001), reflecting a 35.8% reduction in pain. By 60 minutes, the mean VAS score significantly reduced to 2.93 ± 1.61 (p < 0.001), representing a cumulative pain reduction of 61.6% from baseline.

The reductions in VAS scores at both time points were statistically significant, demonstrating the rapid and substantial effectiveness of the intervention in reducing pain among outpatients within the first hour of treatment.

Pain reduction in inpatients

**Figure 2:**
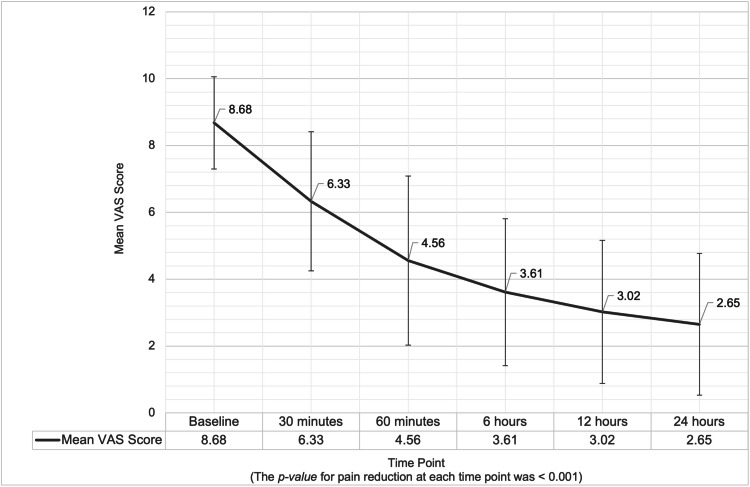
VAS scores over time in inpatients following IM etoricoxib administration Error bars represent SD. The p-value for pain reduction at each time point versus baseline was <0.001. SD: standard deviation, IM: intramuscular, VAS: Visual Analog Scale

VAS scores in the inpatient setting significantly reduced over time. At baseline (0 minutes), the mean VAS was 8.68 ± 1.38. By 30 minutes, the mean VAS decreased to 6.33 ± 2.08, representing a 27.08% reduction (p < 0.001). At 60 minutes, the mean VAS further reduced to 4.56 ± 2.53, reflecting a 47.47% reduction (p < 0.001). This trend continued with a mean VAS of 3.61 ± 2.20 at six hours (58.39% reduction, p < 0.001), 3.02 ± 2.14 at 12 hours (65.19% reduction, p < 0.001), and 2.65 ± 2.12 at 24 hours, indicating a cumulative 69.45% reduction (p < 0.001) (Figure 2).

These findings highlight the significant and sustained efficacy of the intervention in reducing pain among inpatients over 24 hours.

Need for rescue analgesics

Data on the need for rescue analgesics were available for 914 patients (89.8%). Among these, 884 patients (86.8%) did not require rescue analgesics, while 30 (2.9%) needed them. Data were missing for 104 patients (10.2%). The low requirement for rescue analgesics further supports the efficacy of this intervention in managing pain.

Tolerability of IM etoricoxib injection

The tolerability of IM etoricoxib injection was evaluated based on reports of pain at the injection site, allergic reactions, inflammation, and adverse events as recorded by the reporting doctors.

Pain at the injection site

Out of the 1,018 patients, data were available for 975 patients (95.8%). Among these, 590 patients (58.0%) reported no pain at the injection site after 30 minutes. Three hundred thirty-eight patients (33.2%) experienced mild pain comparable to other injections, 45 patients (4.4%) reported moderate pain, and only two patients (0.2%) reported extreme pain. Data were missing for 43 patients (4.2%).

Allergic reactions or inflammation

Data on allergic reactions or inflammation were available for 1,003 patients (98.5%). Among these, 17 patients (1.7%) reported allergic reactions or inflammation, while the majority, 986 patients (96.9%), reported no such reactions. Data were missing for 15 patients (1.5%).

Adverse effects

Adverse events following IM etoricoxib injection were assessed in 1,006 patients (98.8%). Fourteen patients (1.4%) reported adverse events, whereas 992 patients (97.4%) did not experience any adverse effects. Data were missing for 12 patients (1.2%). No serious adverse events were reported in any patient during the study period.

The findings indicate that IM etoricoxib injection is well-tolerated, with most patients reporting no pain or only mild pain at the injection site. The incidence of allergic reactions, inflammation, and adverse experiences was low, further supporting the safety of this intervention.

Physician-reported outcomes on pain relief

Based on physician-reported outcomes, data on pain relief with etoricoxib injection were available for 1,010 patients (99.2%). Physicians reported that 848 patients (83.3%) experienced better pain relief with etoricoxib than with other NSAID injections. Additionally, 145 patients (14.2%) reported equal pain relief, and only 17 patients (1.7%) were noted to have poor pain relief. Data were unavailable for eight patients (0.8%). Physician-reported outcomes strongly indicate that etoricoxib injection provides superior pain relief compared to currently used NSAID injections for most patients, reinforcing its efficacy as observed by the treating physicians.

## Discussion

NICE 2.0 study found that IM etoricoxib led to rapid and sustained pain relief in both outpatient and inpatient settings, with minimal need for rescue analgesia and high physician-reported efficacy. The findings of the NICE 2.0 study reaffirm and expand upon the outcomes of NICE 1.0 by Shetty et al. [12], demonstrating the effectiveness and tolerability of IM etoricoxib in acute pain management across outpatient and inpatient settings. NICE 1.0 focused exclusively on outpatient settings and highlighted significant pain reduction within 60 minutes, with minimal adverse events and tolerability concerns [12]. NICE 2.0 corroborated these findings in outpatients and extended the evidence base to inpatients, providing insights into sustained pain relief up to 24 hours post-injection.

A direct comparison of the percentage reduction in VAS scores at 60 minutes in the outpatient setting highlights key differences. In NICE 1.0 [12], there was a 72.86% reduction in VAS scores at 60 minutes, compared to a 61.6% reduction in NICE 2.0. While both studies demonstrated significant pain reduction, the slightly lower reduction in NICE 2.0 may reflect variations in patient demographics, baseline pain intensity, or clinical environments. Notably, NICE 2.0 involved a more diverse patient population across 296 centers, potentially introducing heterogeneity in pain characteristics and management practices.

The need for rescue analgesics was markedly lower in NICE 2.0 (2.9%) than in NICE 1.0 (12.79%), underscoring greater effectiveness in managing pain without additional interventions [12]. This finding aligns with the broader application of IM etoricoxib in NICE 2.0, which included both inpatient and outpatient scenarios.

Safety and tolerability remained consistent strengths across both studies. In NICE 1.0, 98.69% of patients reported no adverse events, with 56.91% experiencing no pain at the injection site [12]. Similarly, NICE 2.0 demonstrated excellent tolerability, with 58% of patients reporting minimal injection site pain and minimal adverse reactions (1.4%). These results affirm the favorable safety profile of IM etoricoxib, supporting its use in diverse clinical settings.

Physician perspectives further validated the efficacy of IM etoricoxib. In NICE 1.0, 70.23% of physicians rated it as better than currently used NSAIDs [12], while in NICE 2.0, this proportion increased to 83.3%. This improved rating reflects the broader and sustained effectiveness observed in the latter study, particularly in the inpatient population, where extended pain relief up to 24 hours was demonstrated.

Compared to IV diclofenac, the findings further emphasize the advantages of IM etoricoxib in terms of duration of action and rescue analgesic need. According to the Cochrane Review, IV diclofenac achieved a pain reduction of 66% at six hours with a median time to rescue medication of 226 minutes, indicating rapid initial relief but limited long-term efficacy [13]. Haider et al. corroborated these findings, reporting a 63.5% pain reduction at 60 minutes but a significantly higher need for rescue analgesics within four to six hours, ranging between 25% and 30% [14]. In contrast, NICE 2.0 demonstrated a markedly lower need for rescue analgesics, with only 2.9% of patients requiring additional medication, highlighting the sustained efficacy of IM etoricoxib over 24 hours. These differences position IM etoricoxib as a superior option for inpatient pain management where extended relief is crucial, in addition to outpatient settings that require rapid onset of analgesia.

IM etoricoxib and IV diclofenac administration involve practical considerations that influence their usability in clinical settings. IM etoricoxib offers a significant advantage due to its ready-to-use formulation, requiring no dilution or buffering, and is administered via a single IM injection. This simplicity allows for rapid deployment, particularly in outpatient, emergency, or resource-limited settings where establishing IV access can be challenging or time-consuming. Haider et al. emphasized the challenges associated with traditional IV diclofenac formulations, often requiring dilution and slow infusion to mitigate venous irritation [14]. These additional steps could delay the onset of analgesia and introduce risks related to preparation errors. Although newer formulations of IV diclofenac, such as those utilizing hydroxypropyl-β-cyclodextrin, enable bolus injection without dilution, they still depend on IV access, which can pose challenges in specific patient populations or urgent care scenarios. This distinction in administration requirements directly impacts their suitability for pain management, particularly in emergency or resource-constrained environments, where simplicity and speed are paramount.

The study by Shah et al. evaluated a 75 mg/1 mL IM diclofenac formulation for postoperative pain relief in patients undergoing elective surgeries [15]. The baseline VAS score was 7.03 ± 2.15, which decreased by 40.11% at one hour, 61.45% at four hours, and 61.74% at eight hours. By 12 hours, VAS score reduction dropped to 36.42%, indicating diminishing pain relief after eight hours. These results demonstrate significant early pain relief but highlight the need for additional analgesics for prolonged pain management beyond 8-12 hours. These findings, as discussed above, contrast with the current study's findings, which demonstrated sustained pain relief at 24-hour follow-up and a lower need for rescue analgesia.

The current study helps establish the effectiveness and tolerability of IM etoricoxib. However, the limitations of this study include its retrospective and observational design, which may introduce biases related to data collection and analysis. The study relied on medical records, which may only comprehensively capture some relevant clinical details, including patient management and outcome variations. Additionally, the absence of a control group limits the ability to establish causality between the intervention and observed outcomes. Although diverse, the study population may only partially represent all clinical settings or demographic groups, potentially limiting generalizability. Furthermore, the reliance on physician-reported outcomes and patient-reported measures like VAS introduces subjectivity, which might affect the consistency of pain assessment.

## Conclusions

The NICE 2.0 study highlights IM etoricoxib as an effective and well-tolerated intervention for acute pain management in inpatient and outpatient settings. Its rapid onset of action and sustained efficacy, coupled with a minimal need for rescue analgesics, position it as a practical choice for managing acute pain across diverse clinical scenarios. Furthermore, its favorable safety profile, as reflected by the low incidence of adverse events, supports its broad applicability. These findings establish a solid foundation for considering IM etoricoxib as a versatile option in acute pain management. Future prospective, randomized controlled comparative studies are warranted to strengthen the evidence base and explore its efficacy in additional patient populations.
